# PET-MRI effects of N-acetylcysteine in patients with multiple sclerosis

**DOI:** 10.1016/j.ibneur.2026.08.014

**Published:** 2026-08-18

**Authors:** Daniel A. Monti, Sahar Darabi Monadi, Feroze B. Mohamed, Mahdi Alizadeh, George Zabrecky, Nancy Wintering, Monisha Gupta, Alicia Steinmetz, Daniel Kremens, Anthony J. Bazzan, Andrew B. Newberg

**Affiliations:** a Department of Integrative Medicine and Nutritional Sciences, Marcus Institute of Integrative Health, Thomas Jefferson University, Philadelphia, PA 19085, USA; b Department of Radiology, Thomas Jefferson University, Philadelphia, PA, USA

**Keywords:** N-acetylcysteine, NAC, Antioxidant, Multiple sclerosis, PET-MRI, FMRI, Functional connectivity, Positron Emission Tomography, Cerebral metabolism, Cognition

## Abstract

**Background:**

The purpose of this study was to explore if administration of N-acetylcysteine (NAC) in patients with multiple sclerosis (MS) resulted in altered neurophysiological function as determined by the combination of functional connectivity (FC) based on resting Blood Oxygen Level Dependent (BOLD) fMRI and cerebral glucose metabolism using Fluorodeoxyglucose Positron Emission Tomography (FDG PET) and whether those changes were associated with improvements in symptoms.

**Methods:**

Thirty-six patients with mild to moderate MS were randomized to either NAC plus standard of care, or standard of care only. The experimental group received NAC intravenously (50 mg/kg) once per week and orally (500 mg 2x/day) the other six days. Patients in both groups were evaluated initially and after 4 ± 2 months on an integrated PET-MRI platform with resting BOLD fMRI and FDG PET. Clinical symptom questionnaires were also completed at both time points.

**Results:**

The FC data showed significant differences in several brain regions including the inferior frontal gyrus, inferior temporal gyrus, inferior parietal gyrus, insula, cerebellum, and brain stem in the MS group after treatment with NAC. FDG PET demonstrated increased cerebral glucose metabolism in several of these structures including the inferior frontal gyrus and inferior temporal gyrus. Self-reported scores related to cognition and attention significantly correlated with these neurophysiological changes in the NAC group compared to controls.

**Conclusions:**

The results of this study suggest that NAC administration alters resting functional connectivity along with cerebral glucose metabolism in MS patients, and this is associated with qualitative improvements in cognition and attention.

## Introduction

Multiple Sclerosis (MS) is a neurological disorder with a hallmark pathophysiology of white matter injury caused by an autoimmune mediated inflammatory process. Patients can experience one or multiple progressive episodes of worsening MS symptoms which can range across a variety of neurological and cognitive domains (Goldenberg, 2012). Although mediated by immunological processes, the damage to white matter also is thought to be associated with a redox imbalance or oxidative stress (Ibitoye et al., 2016), and a corresponding decrease in the amount and function of antioxidants (Sies and Cadenas, 1985), such as glutathione (Dringen and Hirrlinger, 2003).

Current medical treatments generally target immune aspects of the disease with very few affecting oxidative stress. An exception is dimethyl fumarate (DFM) which is FDA-approved only for adults with relapsing forms of MS, and appears to work by mediating Nrf2-dependent and independent pathways (Yadav et al., 2019). Nrf2 is an important transcription factor that is responsible for maintaining cellular redox homeostasis by increasing the transcription of genes that code for heme oxygenase−1 (HO−1), NAD(P)H dehydrogenase (quinone) 1(NQO1), glutathione-related enzymes, and superoxide dismutase. This results in reduced oxidative stress, increased antioxidant defenses, and the subsequent protection of neurons and oligodendrocytes (Narapureddy and Dubey, 2019). Practically, DFM has important contraindications and monitoring requirements that suggest a need for exploration of other molecules that can function as antioxidants in patients with MS (Xu et al., 2015).

N-acetylcysteine (NAC) has been a consideration to target oxidative stress in MS given its favorable safety profile, though the clinical data remains limited. We previously performed an exploratory study (Monti et al., 2020) that included FDG PET imaging in 24 MS patients receiving a combination of intravenous and oral NAC using the same dosing regimen as in the present study (50 mg/kg IV NAC once per week and concomitant administration of 500 mg oral twice per day). It was found that NAC administration was associated with reported improvements in cognition and also resulted in increased cerebral glucose metabolism on FDG PET in the caudate, inferior frontal gyrus, lateral temporal gyrus, and middle temporal gyrus. Another small study (published as an abstract) of 15 MS patients found that oral NAC given 1250 mg three times per day for 4 weeks improved fatigue symptoms compared to a placebo control (Krysko et al., 2019). A more recent randomized study of 54 MS patients found that oral NAC 600 mg twice per day resulted in decreased lipid peroxidation and improved anxiety symptoms in MS patients (Khalatbari Mohseni et al., 2023).

Administration of NAC has been shown to increase glutathione levels in both animal models (Vina et al., 1983, Martínez-Banaclocha et al., 1997) and human studies (Holmay et al., 2013), and reduce markers of oxidative damage in neurological conditions such as Parkinson’s disease (Martínez-Banaclocha et al., 1999, Khalatbari Mohseni et al., 2023). Directly measuring oxidative stress in the brain is challenging.

One proposed way of assessing brain effects from a treatment like NAC would be functional connectivity assessments, as measured by magnetic resonance imaging (Petsas et al., 2019). It also has been suggested that this could be complemented by measuring changes in cerebral glucose metabolism, as measured by F−18 fluorodeoxyglucose positron emission tomography [FDG PET] (Kreisl et al., 2020). In the present study, we used both technologies and compared them with clinical assessments. Specifically, we utilized resting Blood Oxygen Level Dependent (BOLD) fMRI to assess functional connectivity pre and post intervention, which has been shown to be a promising biomarker for MS (Dogonowski et al., 2016, Faivre et al., 2016, Tahedl et al., 2018).^.^ For an additional level of functional neurological assessment we utilized FDG PET, conducted on an integrated PET-MRI platform. In MS research, structural MRI remains the principal imaging tool for identifying lesions and determining their activity, often through gadolinium enhancement. However, MRI alone provides limited insight into functional or metabolic alterations, and PET-CT does not allow for the simultaneous acquisition of PET and MRI data such as functional connectivity. Thus, PET-MRI offers a combined advantage, including MRI’s ability to explore functional connectivity changes with the ability of PET to characterize metabolic function.

Studies of FC in MS have revealed changes in a variety of structures and networks including the default mode network, salience network, and the frontoparietal network (Bonavita et al., 2011, Rocca et al., 2018, Qu et al., 2026). Such changes in FC are related to the deterioration of the white matter tracts connecting different brain regions. Furthermore, the data correlating FC to cerebral metabolism is also substantial and indicates how excitatory and inhibitory connections can be affected by MS. If NAC is able to improve neuronal function and reduce the oxidative damage to white matter tracts, we anticipate that both FC and cerebral metabolism might be improved. While another fMRI metric, fALFF, relates to fluctuations in neuronal function within a region, there is less evidence in the literature of such alterations in MS and whether those alterations might be correlated with cerebral metabolism. Thus, we have focused on FC and cerebral glucose metabolism for the present study.

In summary, the importance of exploring the impact of NAC on functional connectivity and glucose metabolism is based on several prior findings and factors: 1) There is substantial evidence that FC is abnormal in patients with MS due to its impact on white matter tracts (Kingwell, 2012, Tahedl et al., 2018, Rocca et al., 2022); 2) There is substantial evidence that FDG PET scans reveal abnormal brain metabolism in patients with MS including reductions in a number of cortical areas such as the prefrontal cortex, putamen, supplementary motor area, occipital regions, and pons (Roelcke et al., 1997, Bakshi et al., 1998, Derache et al., 2006); 3) There is evidence that NAC has an impact on neuronal function (Monti et al., 2020, Monti et al., 2025); and, 4) There is a need to correlate changes in neuronal function as assessed by glucose metabolism and functional connectivity.

## Materials and Methods

### Overview

Written informed consent, approved by the Institutional Review Board of Thomas Jefferson University, was obtained from all subjects and the study was registered on clinicaltrials.gov with the following identifier: NCT03032601. Subjects were recruited from local neurology offices and also from the local community through meetings with MS support groups in the area. To be enrolled, subjects had to meet the standard clinical diagnosis of either relapsing remitting or progressive MS along with the following inclusion criteria: Age 21–80 years old and on a stable treatment regimen for at least one month that did not change throughout the treatment period. Patients were excluded if they had any intracranial abnormalities or any medical, neurological (other than MS), or psychiatric disorder that could reasonably be expected to interfere with the assessment of MS symptoms, or with any of the study assessments including the PET-MRI scan results.

To assess symptoms and response to the NAC, patients completed a qualitative evaluation of their symptoms using the 36-Item Short Form Survey, the Mental Health Inventory from the Medical Outcomes Study Survey, and the Perceived Deficits Questionnaire (Ware et al., 1994, Hays et al., 1995). The clinical measures were compared between the pre- and post-intervention time points using a mixed effects repeated measures ANOVA in SPSS and results are reported for the NAC and control group as well as the group*time interaction. Significant clinical changes in the NAC group were also compared to imaging changes using a Pearson R correlation. To determine neurophysiological changes, resting state BOLD MRI imaging was performed simultaneously with an FDG PET scan on an integrated PET-MRI platform at the pre- and post-intervention time points (see detailed neuroimaging methods below).

Thirty-six subjects were enrolled and twenty-nine completed. There were sixteen completers in the NAC treatment group (7 M:9 F; 12 relapsing remitting and 4 primary progressive; mean duration of illness was 15 ± 9; and mean age was 47 ± 15) and thirteen in the waitlist control group (5 M:8 F; 10 relapsing remitting and 3 primary progressive; mean duration of illness was 12 ± 10; and mean age was 51 ± 10). There were no significant differences in the clinical composition of the two study groups.

### NAC Intervention

Subjects were randomized using a permuted block method (using a 1:1 ratio with sealed envelopes for the allocation) either to receive intravenous plus oral NAC or to be placed in the waitlist control condition. We used this dosing regimen based on our prior studies of NAC in neurological conditions including MS (Monti et al., 2020, Monti et al., 2025, Monti et al., 2026). This dosing was originally developed in order to provide a more definitive bioavailability since early data suggested that oral only dosing might not be sufficient. For 4 ± 2 months both groups continued their current standard of care management for MS, with the experimental group receiving NAC in both oral and intravenous form during that same time period. NAC was obtained from the Jefferson Pharmacy as Acetadote (Cumberland Pharmaceuticals). Pharmaceutical grade NAC is an intravenous (IV) medication most used for the treatment of acetaminophen overdose. Doses of NAC were prepared for each patient by a trained study nurse. The dose was 50 mg/kg mixed into 200 ml of D5W infused over approximately one hour 1x per week. Subjects additionally took 500 mg NAC tablets 2x per day on the days that they did not receive the IV NAC. This was continued until presenting for the second PET-MRI scan.

### MRI Acquisition, Analysis, and Statistics

#### MRI Acquisition

The pre and post MRI scans were performed simultaneously with the FDG PET acquisition and included an initial high-resolution structural scan (no additional MRI scans were used in this analysis although all subjects had prior clinical MRIs to confirm the diagnosis of MS). A T1 MPRAGE sequence was used to perform this scan. The main purpose of acquiring structural image is to better examine cortical and subcortical volumes. This was followed by a resting state scan to assess functional connectivity in the different regions of the brain. The scans were performed using a Siemens mMR 3 T PET-MRI scanner using a 12 channel head coil. The T1-weighted imaging parameters are as follows: TR= 1600 ms, TE= 2.46 ms. FOV= 252 mm, Voxel size = 0.5 × 0.5 × 1 mm^3^, slice thickness= 1 mm, and number of slices= 176. The Echo Planar Imaging (EPI) sequence parameters used for resting state BOLD scans are: TR= 2000ms, TE= 30ms, FOV= 236 mm, voxel size = 3x3x4 mm^3^, slice thickness= 4 mm, number of slices= 34. The imaging time for the resting state scan was 8 min and the total imaging time lasted about 45 min.

#### MRI Image Processing and Analysis

To uniquely describe the communication between resting state networks without the influence of noise contaminants, a specialized analysis pipeline is required. This process starts with spatial preprocessing using Statistical Parametric Mapping (SPM) (Wellcome Group, UCL) along with CONN toolbox (Whitfield-Gabrieli and Nieto-Castanon, 2012) which is an open source Matlab (Mathworks, Inc.) based cross platform imaging software. The resting state connectivity analysis pipeline includes a standard preprocessing of fMRI data using CONN and compCor strategy, ROI (Region of Interest) mask creation, time series extraction of ROIs, computing connectivity matrices and first and second level analysis. The preprocessing pipeline used in CONN is as follows: a) Realignment: fMRI data are realigned with the primarily aim of removing motion artefact in fMRI time-series. In this step, the first image is selected as reference and the subsequent images are realigned to the first one using series of rigid body spatial transformation. b) slice-timing correction: corrects the differences in image acquisition time between slices. c) outlier detection. d) co-registration: aligns functional data and the structural data in the same space. e) segmentation of structural MRI in grey matter, white matter and CSF: applies tissue probability map to put the structural MRI in to standard template space. f) Normalization: applies forward deformations from the segmentation step to put the functional data in to standard space. g) smoothing: in this step the signal is averaged to reduce the noise, which helps increasing signal to noise ratio (SNR). This process is performed on all the patients’ pre and post NAC. After the pre-processing is complete, a seed based functional connectivity group analysis is performed. This method assists in finding within group differences through selected ROI that are listed below. Regions of interests (ROIs) are defined and extracted using combined FSL Harvard- Oxford and AAL atlases.

In this study ROI-ROI analysis was used, a subset of SBC (Seed Based Connectivity). FC was considered significant in regions that survived correction for multiple comparisons using the False Discovery Rate Method, (P_FDR<0.05) to measure resting state functional connectivity between subjects before and after taking NAC. The connection is defined as the bivariate correlation between BOLD time series of two ROIs. The target ROIs were selected based on the hypothesized brain regions that are most affected in MS.

### PET-MRI Acquisition, Analysis, and Statistics

#### PET Acquisition

All PET-MRI scans were conducted using a Siemens mMR integrated PET/MRI system (Siemens Medical Solutions USA, Malvern, PA) following standard clinical imaging protocols. These scans were performed initially (the Pre scan) and after the NAC or waitlist control period (the Post scan). For the FDG PET component, an intravenous catheter was placed prior to tracer administration. Participants remained in a quiet environment with their eyes open and ears unobstructed during the procedure. Approximately 185 MBq (5 mCi) of FDG was injected intravenously, and imaging commenced 30 min later. PET data were acquired over a 20-minute interval while concurrent MRI provided anatomical localization and attenuation correction. MRI was also performed for functional connectivity analysis (see below). Upon completion, images were reconstructed in transaxial planes using an iterative reconstruction algorithm.

#### PET Analysis and Statistics

FDG PET images were processed and evaluated using the MIMNeuro analysis platform (MIM Software Inc., Cleveland, OH). The primary analytic metric was regional cerebral glucose metabolism expressed relative to the normative database integrated into the MIMNeuro system. This approach provides a semiquantitative assessment by generating Z scores for each ROI based on comparison with the reference population. All scans were normalized to whole-brain metabolic activity, enabling assessment of metabolic changes between pre- and post-NAC scans as well as comparisons with control participants. Forty ROI-based metabolic measurements, along with clinical endpoints (SF−36, MFIS, MHI, and PDQ), were analyzed using a repeated measures linear mixed-effects (LME) model with correction for multiple comparisons performed using the False Discovery Rate (FDR) approach. The 40 regions were based upon regions that have previously been implicated in MS studies and included the vermis, brain stem, and the left and right caudate, thalamus, insula, cerebellum, and the orbitofrontal, inferior frontal, middle frontal, superior frontal, lateral temporal, medial temporal, inferior temporal, superior temporal, middle temporal, inferior parietal, superior parietal, fusiform, occipital, posterior cingulate, and supramarginal gyrus (SMG).

Proceeding to second-level analysis, as a hypothesis testing step, an independent two sample *t*-test is performed to assess group differences in connectivity. The resting state analysis were done in two different steps. First we considered a more specific analysis accounting for targeted structures of the brain based on those that were observed to be affected on the PET analysis side in order to investigate the connectivity change in those regions and if there were any mutual regions between PET and resting state BOLD analysis. Second, we ran a more stringent analysis applying FDR correction on larger brain areas to assess for additional connectivity changes in the whole brain.

## Results

### Clinical Results

There were no significant differences between the NAC and control group in the pre-intervention period evaluation. Also, there were no differences in the control group between the two time points based on the repeated measures ANOVA. However, in response to the NAC intervention, there were several significant changes in clinical scores both within the NAC group and the across group comparison (group*time). Specifically, there were self-reported improvements in pain, fatigue, and cognitive function sub-scores (see Table 1).

**Table 1:** Clinical measures are shown that were significantly changed in MS patients in the NAC group, between the pre and post the intervention period. Note that there were no significant differences at the pre-time point between the two groups. Also, there were no significant changes in any measures in the control group. The mean value ± standard deviation are presented in addition to the within group p values based on a mixed effects repeated measures ANOVA. Comparison between time*group for the NAC and control group conditions, when using a mixed effects repeated measures ANOVA, were significant for the values in **bold.**

**Test**	**Sub-score**		**Control Group**			**NAC Group**			
			**Pre Mean**	**Post Mean**	**Pairwise p value**	**Pre Mean**	**Post Mean**	**Pairwise p value**	Time* Group p value
**36-Item Short Form Survey**	**Pain**		69.3 ± 21.3	75.7 ± 21.4	0.113	60.2 ± 25.4	69.7 ± 18.8	0.015	0.406
**Modified Fatigue Impact Scale Survey**	**Total**		38.6 ± 22.2	36.1 ± 13.5	0.302	37.1.3 ± 15.8	32.4 ± 15.3	0.016	0.157
**Mental Health Inventory From Medical Outcomes Study Survey**	**Cognitive Function**		69.0 ± 18.1	65.7 ± 13.5	0.524	66.9 ± 20.2	73.1 ± 16.1	0.036	**0.025**
**Perceived Deficits Questionnaire**	**Attention/ Concentration**		8.8 ± 3.8	9.0 ± 3.0	0.800	7.9 ± 4.3	6.5 ± 3.7	0.010	**0.024**

#### Functional Connectivity

The ROI based functional connectivity analysis finds regions of the brain correlated with the activity in a seed region. In our study, we compared the group that took NAC to the control group. In resting state analysis, there were a number of brain regions that showed significant connectivity changes between the NAC treatment and control groups as shown in Table 2. The resting state BOLD analysis found significant changes (FDR corrected for multiple comparisons) in FC particularly with the inferior frontal region, cerebellum, and parts of the temporal and parietal lobes. It should be noted that a number of these areas appeared to be involved in the PET results as well (see Table 3).

**Table 2:** Results show functional connectivity differences in the NAC group compared to controls between the pre and post scans. Results are given for the linear mixed effects model comparison with the T statistic and FDR corrected p value for those regions (the correction is based on the 40 regions targeted in the analysis).

**Structures**	**T Statistic**	**p-FDR**
L Inferior Frontal — L Cerebellum	3.80	0.039
L Inferior Frontal — L Fusiform	−3.88	0.038
L Inferior Frontal — R Cerebellum	4.04	0.036
L Inferior Frontal — R Hippocampus	−4.09	0.036
L Inferior Frontal — Vermis	3.74	0.041
L Inferior Parietal — R Inferior Parietal	−4.17	0.038
L Inferior Parietal — R Superior Parietal	−4.19	0.038
L Inferior Temporal — R Fusiform	−4.64	0.036
L Inferior Temporal — R Occipital	−3.44	0.046
L Middle Frontal — Brain Stem	3.67	0.043
L Middle Frontal — Vermis	3.48	0.045
L Parietal — L Insula	3.98	0.036
L Parietal — R Insula	4.62	0.032
L Prefrontal Cortex — Vermis	4.23	0.038
L Superior Parietal — R Superior Parietal	−3.99	0.037
L Supramarginal Gyrus — Brain Stem	3.91	0.038
R Inferior Frontal — Brain Stem	4.37	0.036
R Inferior Frontal — L Fusiform	−3.88	0.037
R Inferior Frontal — L Hippocampus	−4.38	0.042
R Inferior Frontal — Vermis	3.87	0.037
R Occipital — L Fusiform	−4.26	0.038
R Superior Temporal — R Cerebellum	−3.54	0.045
R SMG — L SMG	−3.97	0.035

(“-“ reflects reduced connectivity between the two structures in the NAC group compared to controls)

**Table 3:** FDG PET results showing brain regions that had statistically significant changes in patients after receiving NAC (p values are FDR corrected for multiple comparisons). These regions had significantly different changes in metabolism when comparing the NAC to the control group when using a mixed effects repeated measures ANOVA.

		**NAC Group**				**Control Group**		
		**Mean Pre**	**Mean Post**	**p value**		**Mean Pre**	**Mean Post**	**p value**
**Caudate**		1.38 ± 1.57	2.02 ± 1.32	0.015		1.65 ± 1.83	1.55 ± 1.90	0.143
**Orbitofrontal Region**		1.75 ± 1.31	2.24 ± 1.07	0.010		2.72 ± 1.11	2.83 ± 0.97	0.235
**Inferior Frontal Gyrus**		0.66 ± 1.05	1.07 ± 1.05	0.017		1.43 ± 0.76	1.34 ± 0.92	0.295
**Fusiform Gyrus**		0.20 ± 0.86	0.91 ± 1.31	0.016		0.30 ± 1.18	0.02 ± 1.10	0.191
**Middle Temporal Gyrus**		−0.17 ± 0.66	0.68 ± 1.08	0.015		0.19 ± 1.11	0.00 ± 1.06	0.265
**Inferior Temporal**		−0.90 ± 1.31	−0.46 ± 0.97	0.040		−0.96 ± 1.10	−0.87 ± 0.87	0.337

Assessing clinical score changes in patients post NAC treatment, there were several correlations found with resting state fMRI connectivity results. Specifically, there was a significant correlations between the change in FC between the right cerebellum and left inferior frontal region with the change in attention/concentration (Pearson R = 0.42, p = 0.028) as well as the change in FC between the left parietal lobe and left insula with the change in total fatigue (Pearson R = 0.41, p = 0.030).

#### FDG PET Cerebral Glucose Metabolism

The FDG PET analysis revealed a number of brain regions that were significantly affected by the NAC intervention (FDR corrected for multiple comparisons; see Table 3). Specifically, there were significant increases in metabolism in the caudate, orbitofrontal region, inferior frontal gyrus, fusiform gyrus, middle temporal gyrus, and inferior temporal gyrus (see Table 3). There were no significant changes observed in the control group.

Finally, we compared correlations between changes in cerebral glucose metabolism measured with FDG PET to the changes in clinical measures and found several significant correlations. These are presented in Table 4.

**Table 4:** Changes in cerebral glucose metabolism that correlated with changes in the self-reported clinical measures (Pearson R).

**Structures – Clinical Measure**	**Pearson R**	**p-value**
Amygdala – Total Fatigue	0.56	0.024
Thalamus – Attention/Concentration	0.57	0.022
Cerebellum – Attention/Concentration	−0.83	> 0.001
Superior Temporal – Attention/Concentration	−0.53	0.037
Fusiform – Attention/Concentration	−0.51	0.041
Inferior Frontal – Pain	0.80	> 0.001
Inferior Temporal – Pain	0.76	> 0.001
Orbitofrontal – Pain	0.69	0.003
Fusiform – Pain	0.73	0.001
Middle Temporal – Pain	0.72	0.002

## Discussion

To our knowledge, this is the first study that evaluated a treatment response to NAC in patients with MS, using the combination of PET and MRI technologies. The results suggest that there were several brain regions that underwent significant changes in their FC and glucose metabolism in response to the NAC. These functional brain assessments combined with clinical data provide new information on the neurophysiology of MS and potential effects of NAC as a treatment.

In the present study, the experimental group showed that some brain regions had decreased FC in response to treatment and some areas had increased FC, suggesting NAC may enhance FC in areas in which it is impaired, and improve FC in areas in which it is abnormally heightened. This is consistent with what is known about the balance between inhibitory and excitatory processes, both of which can be affected by the lesions of MS (Stampanoni Bassi et al., 2017; Logothetis, 2002). In general, excitatory glutamate-mediated synaptic activity generates local field potentials that drive metabolic demand and cerebral blood flow, whereas inhibitory GABAergic interneurons affect the timing, synchronization, and gain of neuronal firing across distributed networks (Lauritzen et al., 2012). Consequently, functional connectivity is increasingly viewed as a macroscopic manifestation of the dynamic balance between excitation and inhibition (E/I balance), rather than simply reflecting overall neuronal firing rates (Xu, 2015). Disruption of this balance alters the temporal coherence of neuronal oscillations and is believed to underlie the abnormal network connectivity observed in numerous neurological disorders such as MS (Gao et al., 2018, Zhang et al., 2024).

Recent multimodal imaging studies combining proton magnetic resonance spectroscopy (1H-MRS) with fMRI have further demonstrated that regional concentrations of glutamate and GABA are associated with both resting-state and task-related BOLD activity. Higher cortical GABA concentrations are generally associated with reduced local BOLD responses and decreased functional connectivity, whereas greater glutamatergic activity is often associated with stronger activation and enhanced communication across distributed brain networks (Duncan et al., 2014). These observations support the concept that functional connectivity reflects the integrated effects of excitatory and inhibitory neurotransmission on large-scale neuronal networks. Because disorders such as multiple sclerosis are associated with disturbances in glutamatergic signaling, GABAergic inhibition, oxidative stress, and mitochondrial dysfunction, resting-state functional connectivity may be an important biomarker of neuronal network integrity and neuroplasticity that complements metabolic findings from FDG PET.

Key areas of the brain with altered FC such as the fusiform gyrus, middle temporal gyrus, and inferior frontal regions also had increased metabolic activity observed on FDG PET. Thus, the findings of both altered FC and increased glucose metabolism in the temporal lobe and inferior frontal region along with noted improvements in cognitive processing and attention, cumulatively suggest a potential specific effect of NAC treatment that is encouraging and warrants further investigation. Impaired cognition is a significant problem among patients with MS with between 40% and 70% of patients affected (Chiaravalloti and DeLuca, 2008, Lovera and Kovner, 2012, Benedict et al., 2020). Memory, information processing speed, and attention and executive function, also are commonly affected in MS patients (Miller et al., 2018), significantly impacting quality of life (Hoogs et al., 2011, Gómez-Melero et al., 2024).

There is one study in which FDG PET data has been used to evaluate a therapeutic response in MS patients. However, this study focused on white matter glucose metabolism in 33 patients with MS and evaluated changes related to several different therapies. It reported a mixed response and no clear difference between treatment arms, but this might have been due to the limited sample size.

The current study specifically explored changes in cortical glucose metabolism since some studies have found that severity of MS is associated with worse cortical glucose metabolism (Blinkenberg et al., 1999, Mathur et al., 2014). Thus, the increased metabolic activity observed in response to NAC may suggest improvement in brain function.

This study utilized intravenous NAC as the primary intervention, supplemented by oral, based on the limited available data. Oral absorption is variable, which is likely the reason that infusions alone or the combination of infusions plus oral has been the most used in neurological studies. For example, one MRS study showed an increase in brain glutathione levels in patients receiving an intravenous dose of NAC (Tommasin et al., 2019), but another MRS study using only oral NAC revealed no increase in brain glutathione (Coles et al., 2017).

There are several important limitations to the current study. Although we included a control group, it was not a placebo group and this was not a blinded study. It is possible that the improvements that we observed in the NAC group could have been influenced by a placebo effect. In addition, a more likely effect given the lack of blinding is that patients may have answered the self report clinical measures in a manner more suggestive of a beneficial effect in patients who knew that they were receiving NAC. However, given the ethical concerns raised regarding giving MS patients a placebo for weekly IV infusions (Polman et al., 2008), we opted to have a waitlist control for this initial study. The treatment was offered without charge after the experimental period. Future studies would be strengthened by formal neuropsychological testing. To better elucidate possible mechanisms of treatment effect, it also may be helpful in future studies to measure NAC or glutathione levels in the brain either through direct measurement of CSF or through an approach such as MR spectroscopy. We also anticipate that future studies can utilize more advanced MRI sequences with longer acquisitions to improve reliability of the data on FC parameters. Another limitation was the method for evaluating the FDG PET scans. While we used a commercially available FDA approved software system, it could still only evaluate relative effects in brain regions which are normalized against the whole brain metabolism. Unfortunately, the only way to truly evaluate whole brain metabolic changes (or large brain areas that might be affected in MS), as well as regional changes, would be to perform arterial sampling to obtain absolute quantification of glucose metabolism. This requires a much more complex approach to imaging the patient that typically includes arterial sampling. While larger areas may be affected in MS, due to the additional heterogeneity of the disease, it may be difficult to find statistically significant results in general. However, it is noteworthy that there were a select group of specific regions found to be involved in the metabolic response to NAC. Another important question is that although this study provided patients with both intravenous and NAC, it is not known whether this combination is required to produce clinical or physiological effects in MS, and what would be the ideal treatment length. Finally, clinical results are based on self-reported questionnaires.

In summary, this is the first study to use PET-MRI to evaluate the effects of NAC administration in patients with MS. The data suggests a potential role for FC and cerebral glucose metabolism to help characterize disease characteristics and elucidate the clinical effects of MS treatment. Importantly, the current study provides encouraging initial data that NAC might positively impact resting FC and cerebral glucose metabolism in MS patients, and this appears to be associated with improved symptoms, particularly related to cognition and attention. The next level of inquiry with a larger randomized, double blind, placebo-controlled efficacy trial is warranted.

### Ethics approval and Consent to participate

The study was performed in accordance with the Declaration of Helsinki and was approved by the Institutional Review Board at Thomas Jefferson University.

## Authors’ Conflict of Interest

No authors have any financial conflict of interest related to the study.

## Consent for Publication

Not applicable.

## Trial Registration

Registered on clinicaltrials.gov 01/26/2017, NCT03032601

## CRediT authorship contribution statement

**Nancy Wintering:** Writing – review & editing, Supervision, Project administration, Methodology, Investigation, Conceptualization. **Monisha Gupta:** Writing – review & editing, Project administration, Investigation. **Mahdi Alizadeh:** Writing – review & editing, Methodology, Formal analysis. **George Zabrecky:** Writing – review & editing, Methodology, Funding acquisition, Conceptualization. **Sahar Darabi Monadi:** Writing – original draft, Methodology, Formal analysis, Data curation. **Mohamed Feroze B:** Writing – original draft, Resources, Methodology, Conceptualization. **Newberg Andrew B:** Writing – original draft, Supervision, Project administration, Methodology, Investigation, Formal analysis, Conceptualization. **Monti Daniel:** Writing – original draft, Supervision, Project administration, Methodology, Investigation, Funding acquisition, Formal analysis, Conceptualization. **Daniel Kremens:** Writing – review & editing, Methodology, Conceptualization. **Alicia Steinmetz:** Writing – review & editing, Project administration, Investigation. **Bazzan Anthony:** Writing – review & editing, Methodology, Conceptualization.

## Declaration of Competing Interest

None of the authors have any conflicts of interest nor competing interests to report. This paper is our original work. We certify that all authors named deserve authorship, and that all authors have agreed to be so listed and have read and approved the manuscript.

## Data Availability

The datasets used and/or analyzed during the current study are available from the corresponding author on reasonable request.
